# Bistable Cell Fate Specification as a Result of Stochastic Fluctuations and Collective Spatial Cell Behaviour

**DOI:** 10.1371/journal.pone.0014441

**Published:** 2010-12-28

**Authors:** Daniel Stockholm, Frédérique Edom-Vovard, Sophie Coutant, Peggy Sanatine, Yoshiaki Yamagata, Guillaume Corre, Laurent Le Guillou, Thi My Anh Neildez-Nguyen, Andràs Pàldi

**Affiliations:** 1 Généthon, Evry, France; 2 INSERM U951, Université Evry Val d'Essonne, Evry, France; 3 UMR951, Ecole Pratique des Hautes Etudes, Evry, France; 4 LPNHE - Université Paris 6, Bureau 412 - Tour 43 RdC, Campus de Jussieu, Paris, France; University of Oxford, United Kingdom

## Abstract

**Background:**

In culture, isogenic mammalian cells typically display enduring phenotypic heterogeneity that arises from fluctuations of gene expression and other intracellular processes. This diversity is not just simple noise but has biological relevance by generating plasticity. Noise driven plasticity was suggested to be a stem cell-specific feature.

**Results:**

Here we show that the phenotypes of proliferating tissue progenitor cells such as primary mononuclear muscle cells can also spontaneously fluctuate between different states characterized by the either high or low expression of the muscle-specific cell surface molecule CD56 and by the corresponding high or low capacity to form myotubes. Although this capacity is a cell-intrinsic property, the cells switch their phenotype under the constraints imposed by the highly heterogeneous microenvironment created by their own collective movement. The resulting heterogeneous cell population is characterized by a dynamic equilibrium between “high CD56” and “low CD56” phenotype cells with distinct spatial distribution. Computer simulations reveal that this complex dynamic is consistent with a context-dependent noise driven bistable model where local microenvironment acts on the cellular state by encouraging the cell to fluctuate between the phenotypes until the low noise state is found.

**Conclusions:**

These observations suggest that phenotypic fluctuations may be a general feature of any non-terminally differentiated cell. The cellular microenvironment created by the cells themselves contributes actively and continuously to the generation of fluctuations depending on their phenotype. As a result, the cell phenotype is determined by the joint action of the cell-intrinsic fluctuations and by collective cell-to-cell interactions.

## Introduction

Phenotypic heterogeneity is an intrinsic feature of many cell lines [1], [2], [3], [4], [5]. This heterogeneity could be simply due to the stochastic variations at the level of gene expression or protein synthesis [6], [7]. However, the phenotype of the individual cells in these populations is not constant. The cells fluctuate slowly but continuously between different phenotypic states that leads to a dynamic equilibrium with relatively constant proportions of various phenotypic variants in the population. Theoretically it is possible to explain the population-level stability solely as the reflection of the bi- or multistable cell-intrinsic fluctuations of the gene expression in individual cells where a given phenotype would correspond to a metastable state of the fluctuating transcriptome [8], [9]. In this case, the proportion of a given phenotype would reflect the probability of an individual cell to reach that phenotype. Alternatively, cell-to-cell interactions between the cells in the population can influence the noise dynamics of each individual cell either by modulating the noise in general or by increasing or decreasing the probability to reach a given phenotypic state. In the present study, we set out to investigate the second hypothesis.

An obvious and well-known manifestation of the non-genetic cell individuality in culture is the unique migration properties of each cell. Migration can induce fluctuations of local cell density and create spatial arrangements at the population level. It is likely that intracellular fluctuations and variations in cell-to-cell interactions may interfere in a non-trivial way. Very little is known about the outcome of these interactions and their potential role in cell fate decisions. We have previously observed that cell density can increase the gene expression noise and induce epigenetic effects leading to stable changes in gene expression [10]. We have also observed that cells with stem-like characteristics tend to appear in low density regions of myogenic cell populations [1] suggesting that the fate choice between a stem cell-like and a differentiation committed phenotype is controlled by the appropriate local microenvironment generated by the cells themselves.

In the present study, we investigated the relationship between the phenotypic switch and spatial distribution in clonal populations of primary muscle-derived cells using cell culture experiments and computer simulations. We show that proliferating myogenic cells in culture can fluctuate between phenotypic states under the effect of the local microenvironment. Computer simulations suggest that the phenotypic fluctuations follow a bistable dynamics driven by a microenvironmental context-dependent intracellular noise. The microenvironment is shaped by the cells themselves because their motion generates non-random cell interactions. In this way each cell contributes to put together its own microenvironment that in turn stimulates the fluctuation between the phenotypes until a state with low noise is found.

## Results

### Phenotypic heterogeneity of the primary human myoblasts

**Figure 1:**
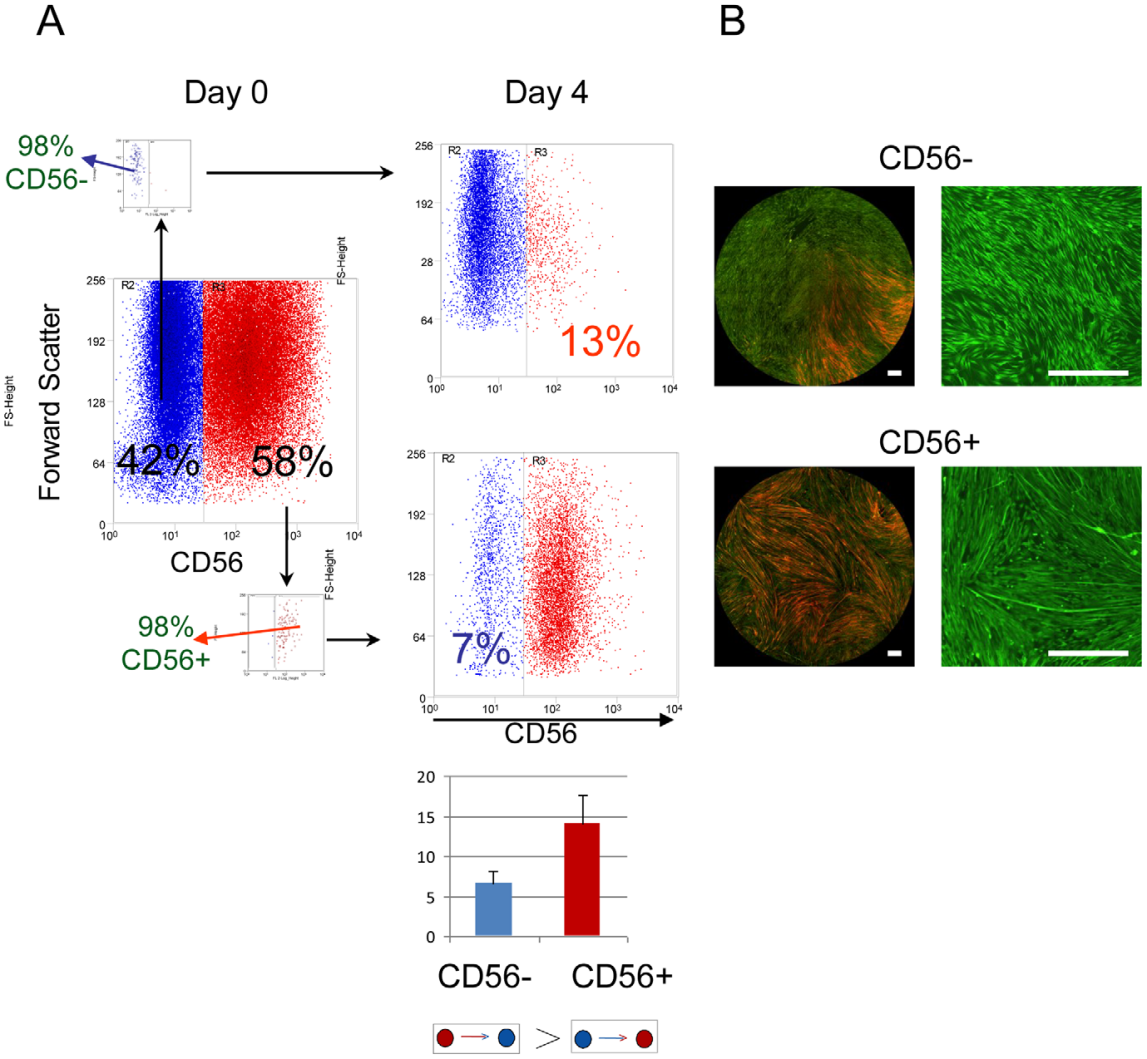
Reversibility of the CD56+ phenotype. **A**. Flow cytometry analysis of the human myoblast population typically shows a 1/3 ratio CD56−/CD56+ cells (Day 0, left). When sorted, CD56− cells change their phenotype more frequently than the CD56+ cells as shown by the higher proportion of converted cells 4 days after sorting (right panel). The small insets represent the controls for cell sorting efficiency. The proportions represent the average of at least three independent experiments. **B**. Low resolution micrographs of the immunostaining of whole populations (CD56+ cells in red) show (left panel) that cloning of individual CD56− or CD56+ cells resulted in mixed populations with a higher proportion of converted cells in the clones derived from CD56− cells. Myoblast populations derived from sorted cells were cultured for 7 days. High resolution pictures (right panel) show the emergence of wave-like spatial patterns in both cell populations. The formation of myotubes is only observed in the originally CD56+ population. Both cultures were stained with CFSE (green fluorescence) for better visibility. Scale bar  = 200 µm.

We used populations of primary mononuclear cells isolated from human muscle [11] that contain progenitor cells with high proliferative capacity that are usually considered as definitively committed to muscle fate. These cells express myogenic markers believed to specify definitive cell commitment such as CD56 (NCAM) [12]. At high density, the cells become elongated, align with each other and form typical wave-like structures. At confluence, the aligned cells fuse to form myotubes. In a typical growing population, 30 to 40% of the proliferating cells do not express CD56 and are usually considered as “contaminating” fibroblasts [12]. In order to elucidate whether these two subpopulations represent two distinct phenotypes or two stages of the myogenic differentiation process we separated the CD56+ and CD56− cells using a cell sorter and cultured them separately. Both subpopulations proliferated at about the same rate, reached high density simultaneously and produced wave-like spatial arrangements typical for myogenic cells (Fig. 1 right panel). In spite of these similarities, the two cell fractions displayed fundamental functional differences. At high density, cells expressing CD56 readily fused to form myotubes, while only a few myotubes were observed in the population of CD56 negative cells (Fig. 1 right panel). In order to show that the difference between the two populations cannot be reduced to the simple ectopic silencing or activation of the CD56 gene, we have investigated the CpG methylation pattern of the gene. As explained in Supporting Document S1, there was no difference in the methylation pattern.

Next, we sorted by flow cytometry individual CD56+ and CD56− cells and cultured them separately for 14 days. The immunochemical analysis of the resulting clonal populations (more than 100 clones) showed that they contained both CD56+ and CD56− cells. Since all cells in a clone derived from either a CD56+ or a CD56− founder, the simultaneous presence of both + and − cells in the populations indicates that the cells are able to change phenotype. In order to determine the frequency of these phenotypic switches, the subpopulation of the two cell types were sorted from the original mixed population and cultured separately under similar conditions. Four and seven days later, the proportion of CD56+ and CD56− cells was determined by flow cytometry. The results show (Fig. 1 left panel) that the proportion of the cells with opposite phenotype increased constantly in both the initially CD56+ and the CD56− cell populations. This observation shows that the phenotypic interconversion is relatively frequent in these cells and occurs continuously. As a result, the simultaneous presence of the two phenotypes in the growing population is the result of a dynamic equilibrium of the two opposite processes. Importantly, there were proportionally less CD56+ cells in the initially CD56− population than CD56− cells in the originally CD56+ culture (Fig. 1B) suggesting that the transition of the CD56− cells into CD56+ occurs less frequently than the opposite. This is counter-intuitive if we consider that the majority of the cells were CD56+ in the original population and this latter state is usually considered as definitively committed. A possible explanation for this apparent contradiction could be that the rate of phenotypic switches is not a simple cell autonomous probabilistic event, but may depend on some features of the population as a whole.

### Influence of the cellular microenvironment on the phenotype

**Figure 2:**
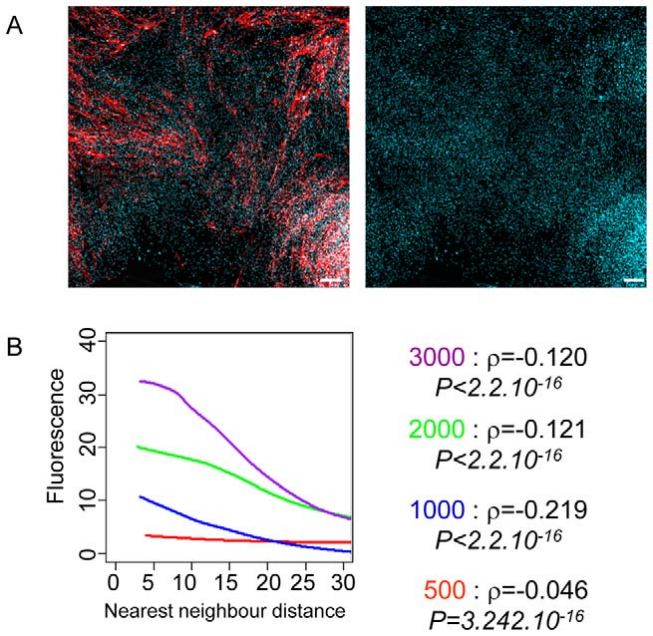
Spatial pattern of CD56+ cell distribution in unperturbed myoblast cultures after 7 days. A. Red staining shows the CD56+ cells detected with a fluorescent monoclonal anti-CD56 antibody (left panel) and blue shows cell nuclei using DAPI staining. The cell-density differences are clearly visible when nuclear DAPI staining is shown separately (right panel). Scale bar  = 100 µm. B. Locally weighted scatter plot smoothing (LOWESS) regression analysis (left panel) shows that the spatial distribution of the CD56 expressing cells is correlated with the local cell density at all 4 global initial densities examined (red, blue, green and violet lines). Distance to the closest neighbour is used as a measure of local cell density. The CD56 fluorescence is the highest at small neighbour distances ( = high density). The Spearman's rank correlation ρ is relatively modest, but statistically highly significant at all global densities (right panel).

Therefore, we investigated whether global characteristics of the culture such as cell density may influence the ratio of CD56+ and CD56− cells. In a typical experiment, the cells were cultured at the initial density of 500, 1000, 2000 and 3000 cells/cm^2^ for 6 days, then fixed and immunostained with a CD56 antibody. We scanned the whole population microscopically at high resolution and recorded the position and the fluorescence intensity of each cell. We found that the proportion of cells with CD56 labelling above the background level increased with the global cell density of the culture: we observed 60%, 68%, 83.6% and 87% CD56+cells at the 4 different densities, respectively. Importantly, these differences are not due to the differences in the growth phase of the populations with different starting cell densities, because the total cell numbers suggest a similar number of cell divisions in all four cases (14250 cells/cm^2^, 17450 cells/cm^2^, 33240 cells/cm^2^ and 62380 cells/cm^2^ in the four cultures respectively). It is clear that at high density there are proportionally more CD56+ cells than at low density. This conclusion is supported by the fact that even within the same culture the spatial distribution of + and – cells was different. Cells with high CD56 levels appeared to be concentrated in high local cell density regions and were less frequent in low-density regions (Fig. 2A). By contrast, CD56− cells were less frequent in high-density regions and prevailed in low-density regions of the culture. To evaluate statistically the validity of this observation, we plotted the CD56 fluorescence intensity measured for each cell in the population as a function of the distance to the closest neighbour as an estimator of the local cell density. The smaller these distances, the higher is the local density. We used locally weighted scatterplot smoothing (LOWESS) regression analysis to visualize the correlation between these two parameters. This analysis confirmed that the spatial distribution of the CD56 expressing cells was non-random and correlated with the local cell density in all cases (Fig. 2B). Cells with a high expression level tended to be located in regions of high local cell density. Overall, the correlation was relatively modest, but statistically highly significant as determined by the Spearman's rank correlation test (Fig. 2B). The negative value of the correlation coefficient ρ indicates a positive correlation between the density and the fluorescence. The strongest correlation was observed at an intermediate global density where the differences in local densities between different parts of the population were substantial. The correlation between the spatial distribution and cell phenotype points to the importance of the microenvironment and intercellular interactions in the cell fate determination. Nevertheless, even highly dense regions contain many low CD56 expressing cells, suggesting that the mechanistic link between the local cell density and the cell phenotype represented by the CD56 expression level is not simple and linear.

These observations raise the question of how cells are able to “sense” local cell density. One possibility is that the sensing is contingent on the diffusion-dependent local concentration of molecules secreted to or depleted from the medium. Whatever the exact chemical nature of these molecules, the cells would then respond in a paracrine or autocrine fashion. The concentration of such molecules is expected to vary as a function of the cell distribution in the culture and form local concentration gradients depending on the local cell density.

**Figure 3:**
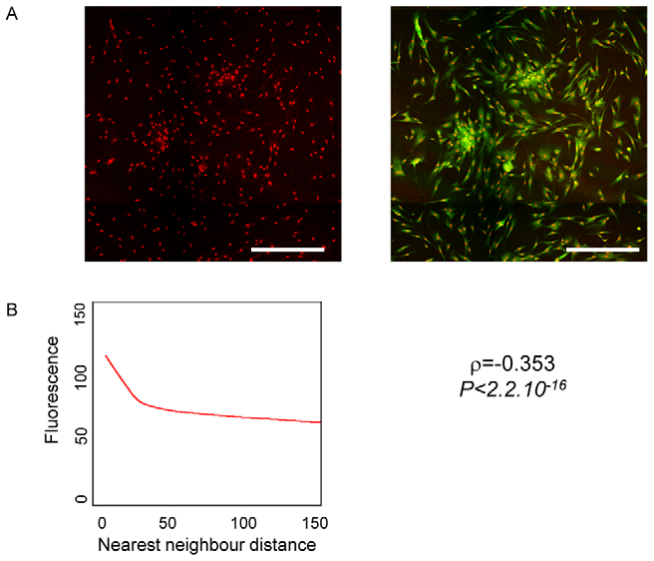
The intensity of superoxide anion labelling in a typical myoblast culture with fluctuating local cell densities is higher in cells of high-density regions as confirmed by the fitting of the LOWESS curve to the scatter plot of fluorescence intensities as a function of minimal neighbour distances. The Sperman's rank correlation ρ was highly significant. Scale bar  = 100 µm.

In order to investigate whether cell density dependent concentration fluctuations can influence cell physiology we investigated the stress-response of the cells. The intracellular superoxide anion concentration was measured in the cells as described in the Materials and Methods section and correlated to the local cell density using the same approach as in the case of CD56 expression. The intensity of superoxide anion labelling is higher in cells of high density regions as confirmed by the fitting of the LOWESS curve to the scatterplot of fluorescence intensities as a function of minimal neighbour distances. The Sperman's rank correlation ρ was highly significant (Fig. 3). This observation clearly shows that the cells sense the high local density and produce a stress response to it. Although it does not demonstrate that the superoxide anions are mechanistically involved in the phenotypic switch, it is possible that the stress response contributes to the initiation of the phenotypic switch. Previous observations showed that increased cell density can indeed lead to the increase of phenotypic heterogeneity by directly acting on gene expression noise [10].

Overall, these observations indicate that the primary myogenic cells show dynamic phenotypic plasticity in culture that allows them to switch between at least two distinct phenotypes: one characterized by the expression of a strong myogenic cell surface molecule CD56 and able to fuse into myotubes and the second by the absence of this marker and a low propensity to fuse. Nevertheless, the two cell categories share several properties, such as the capacity to proliferate and form wave-like patterns. The transition rate between the two states is relatively low and, unexpectedly, CD56+ cells switch to CD56− state more easily than the opposite. In addition, the spatial distribution of the CD56+ cells is non-random: they preferentially accumulate in regions with high local cell density.

### Modelling the phenotypic switch

**Figure 4:**
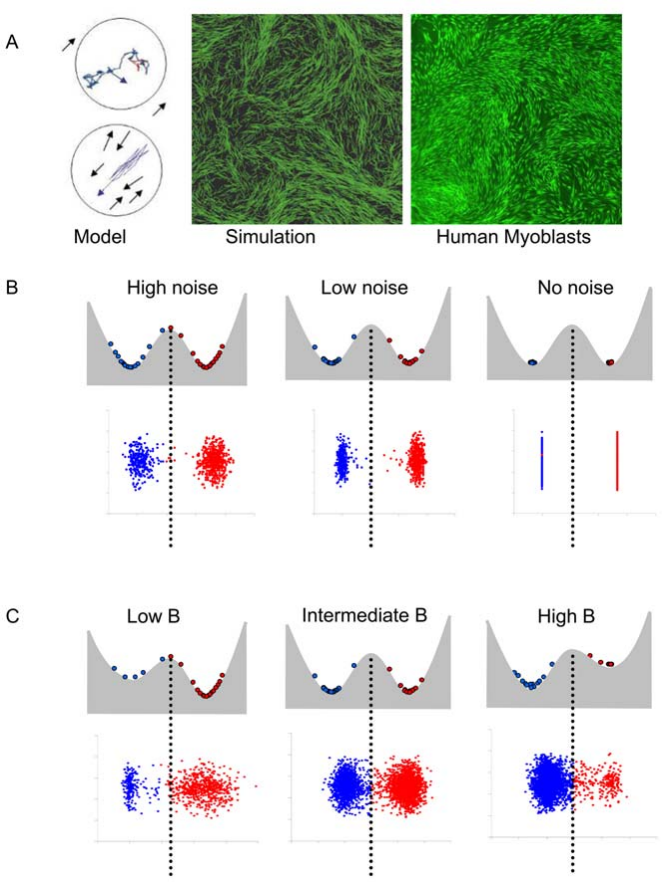
Theoretical model of cell alignment and bistability. A. Schematic representation of the basic rules for the migration and alignment of the cells depending on the presence of a neighbour within reach (left panel). Solitary cells move randomly (upper part). Neighbours within a circle of a given radius adjust their migration direction (lower part). Snapshot (middle panel) of a simulated cell population shows wave like spatial pattern similar to living cells (right panel; the cells were stained with a green membrane stain CFSO for better visibility). B. Schematic representation of the role played by the noise (B) and the regulation of the kinetics (C) of the transition between the two states in a bistable system. The two stable states represented by the two wells. The frequency of transition from one state to the other is determined by the level of noise ( = stochastic fluctuations in gene expression) (B), but the number of cells in each state at equilibrium depends only on the shape and depth of the wells ( = kinetic parameter B of the system) as shown on C.

The co-existence of two of phenotypically distinct subpopulations suggests that the phenotypic conversion of individual cells follows a bistable dynamics. A cell can be considered as bistable if under the same conditions it can adopt one of two different and stable phenotypes with the intermediate states being unstable. Bistability may arise from the internal dynamical properties of gene networks that bring about the phenotype. Although gene regulatory networks are usually complicated, in the simplest cases a single regulatory loop is sufficient to allow two stable alternative states, attractors, with different active and silenced genes. The probability of a cell with bistable properties to adopt one or the other phenotypic state is specified by the regulatory parameters of the system, more specifically by the threshold separating the two stable states [13], [14]. However, the transition between the states is triggered by the noise arising from the stochastic nature of molecular interactions (Fig. 4B) and the frequency of the phenotypic switches is dependent of the noise level. As a result, in a population of bistable cells, the proportion of the two possible phenotypes reflects the regulatory properties of the underlying gene network, while the velocity to reach the phenotype depends on the noise level. The systematically observed high CD56+/CD56− cell ratio in the myoblasts suggests that the equilibrium between the two possible states is biased and the cells are more prone to become CD56+. However, the observation that CD56+ cells relaxed faster to the CD56− phenotype than the opposite contradicts this. In addition, the bistability of the individual cells cannot explain their non-random spatial localization within the population. This is only possible if the cells can sense the local cell density and respond to it by changing their phenotype.

In order to understand how the generic principles of bistability and the capacity of sensing the local cell density bring together the dynamical properties observed in our muscle derived cell system we performed computer simulations. The aim of the simulations was to produce qualitative rather than quantitative predictions on the behaviour of the system. We focused our attention on the effect the cell density may have on the regulatory parameters of the bistable phenotypic transition, on the noise that triggers the change and the possible impact of the spatial patterns formed by the cells. We first designed an agent-based model that faithfully reproduced the formation of regions with variable cell densities and wave-like alignments observed in myoblast cultures. Next, we integrated in this basic model the capacity of bistable phenotypic switch in individual cells that depends on the sensing the local and global cell density through the consumption of a diffusion-limited substance in the environment. Since the exact nature of the regulatory network underlying the phenotypes of our cells is unknown, our model focuses on the generic properties of the system rather than a numerically accurate and detailed description. The effect of cell density on the frequency and localisation of high and low *P*-producing cells as a function of density-dependent regulation, intrinsic and cell-context dependent noise modulation was investigated in cell populations with or without cell alignments.

#### Simulating pattern formation as a result of collective movement

The migration and proliferation properties of the cells were implemented in an agent-based model based on detailed time-lapse observations of living cell cultures as described earlier [1]. Briefly, we used time-lapse movies to obtain a large number of data on the direction and velocity of migration. We have quantified the cell velocities and the directions of the movement by comparing the positions of the cell nucleus on each image of a time sequence (Fig. S1A and B). The cell cycle length was calculated based on individual cell tracking and cytometric analysis of cell proliferation (Fig. S1C). The observations of the time-lapse movies revealed that the cells start to align their movement when the local density becomes high. The initially random two-dimensional cell motion becomes progressively random in a single dimension. Even at high density, the cells move along each other and change direction apparently randomly. The multinucleated cells derived from cell fusion also move in this way. Therefore, the ordered spatial cell patterns seen on fixed cell populations are in fact very dynamic; they are based on fluctuating individual cell motions constrained by the high density. Since the emergence of this pattern is reminiscent of the flocking of birds, fishes or microorganisms, we used the same logic to simulate the dynamic behaviour of myoblast: at low density, the cells move randomly, but at high density, they align their movement with the close neighbours. The direction and velocity of the motion remains independent of the neighbours (Fig. 4A). These simple rules were sufficient for the emergence of a collective movement of the neighbouring cells. A snapshot of such a population is a spatial pattern similar to that observed in myogenic cultures (Fig. 4A, middle and right panels). Because of the high heterogeneity of local densities, a proportion of cells conserved their independent random migration behaviour both in living and simulated cell populations.

In order to determine whether the collective movement of the cells influences their phenotypic change we created an alternative version of the model in which the migration of the cells was not constrained by the cell density. The bistability of phenotypic states and the capacity of density sensing were identical in the two versions of the model.

#### The effect of the local cell density on the bistable phenotypic switch

In order to study the effect of local cell density on the phenotypic fluctuation we integrated the bistability of phenotypic transition in individual cells with the agent-based model simulating the pattern formation. We simulated the bistable phenotype transition as a process of “production” of the new phenotype “high *P*”with sigmoid kinetics described by the Hill function and a setback to the “low *P*” phenotype that followed linear kinetics. The non-linear production and linear degradation together ensure the capacity of dynamic bistable behaviour for every cell (see Materials and Methods for the exact function incorporating the Hill function for the production and linear function for the degradation). We assume that during the *in silico* experiment all cells tend to a final equilibrium of “high P” and “low P”phenotypes of approximately 3 to 1, as observed in living cell experiments. A cell in the model was considered “high *P*” if the actual production rate of *P* was higher than the rate at the inflection point of the Hill function.

The control parameters in the model were: (1) *C_B_* that determines how *P* production is dependent on local cell density by setting the threshold between the two stable states: (2) noise that can be considered as a measure of stability. Typically, the noise term is defined [15] by its relationship with the mean (here *P*). In our model, the noise term was dissociated into two independent terms: intrinsic noise (*N_int_*) that depicts a white noise that occurs in any cell and is independent of the cell density and cell phenotype and context- or density-dependent noise (*N_ext_*) that is a function of local cell density and the cell phenotype. *N_ext_* was high in “low *P*” cells in high local density regions and in “high *P*” cells in low-density regions but low in “low *P*” cells in low local density regions and in “high *P*” cells in high-density regions (see the Materials and Methods section and Fig. S4 for the exact function describing *C_B_* and the noise terms). In other terms, two cells with identical levels of *P* are characterized by different level of noise i.e. different stability depending on whether they are located in high- or low-density regions.

The cells in the model were able to sense the local density through the detection of the concentration of a diffusible molecule *R*. The value of *R* can be considered as a measure of local cell density. The actual concentration of *R* was the result of a dynamic equilibrium between the uptake and consumption by the cells and the replacement by diffusion from the culture medium. In high cell density regions, the uptake exceeds the diffusion resulting in a reduction of the local concentration of *R*. Although we used the oxygen concentration and the reaction of the cells by a hypoxic stress as an example, we do not make any explicit hypotheses about the chemical nature of the diffusible substance.

**Figure 5:**
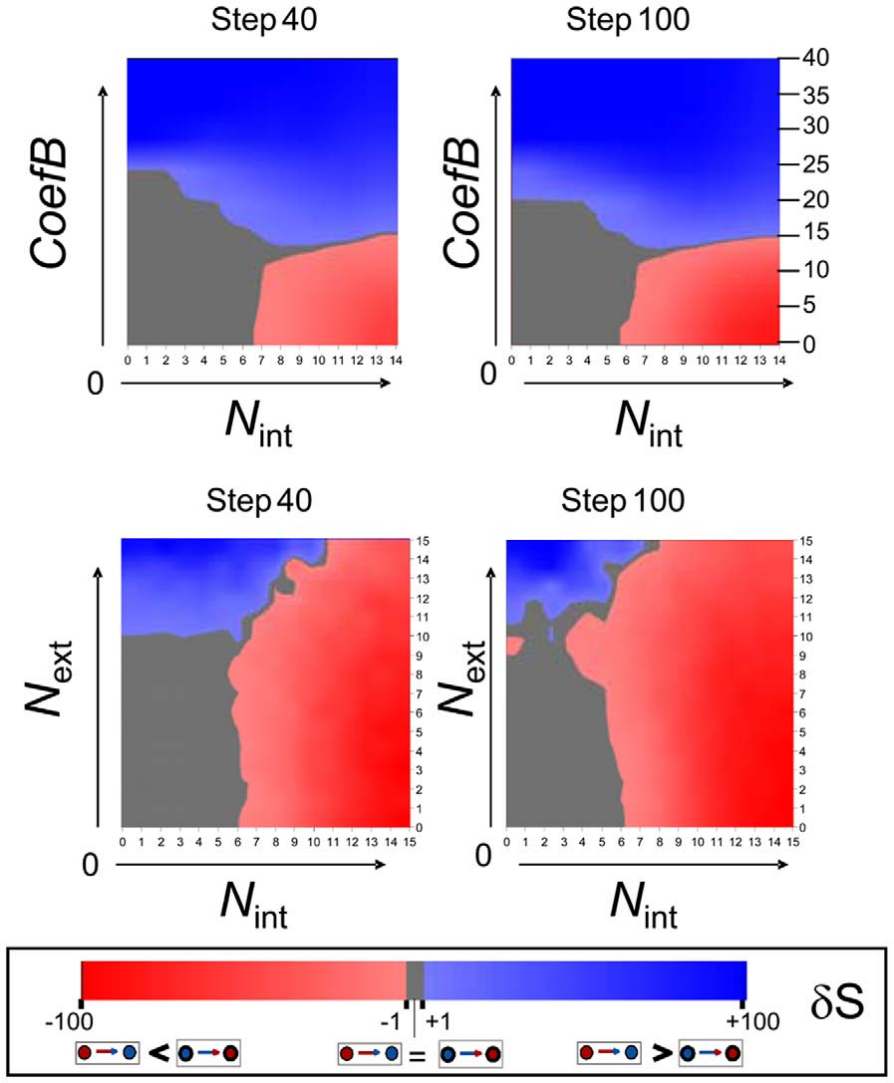
Analysis of the parameter space. The difference in the conversion rates δS of sorted “high *P*” and “low *P*” cells is represented in the parameter space defined by either the context dependant regulatory Coefficient *C_B_* and the intrinsic noise (*N_int_*) or by the context-dependent noise (*N_ext_*) and intrinsic noise (*N_int_*) parameters at the steps 40 and 100 of the simulation. The three possible regimes are indicated by a colour code. The area in blue (high context dependent and low intrinsic noise) indicates the part of the parameter space with conversion rates higher for the “low *P*” cells, while in the area indicated in red (high intrinsic noise) the opposite tendency prevails. The area in grey indicates the regime of low noise where no conversion occurs.

We explored the parameter space defined by either *C_B_* and *N_int_* or *N_ext_* and *N_int_.* Since it is not possible to determine experimentally neither the exact rate of *P* production nor the level of noise in living cells, the model produced only qualitative predictions. We were interested in the range of values that allowed bistability and resulted in changes that could be considered as biologically plausible. The simulations started with a small number of either CD56− (“low*P*” cells in the model) or CD56+ (“high*P*”) cells and reached the maximal population size of about 5000 cells after 150 to 200 steps. This setup is the *in silico* equivalent of the cell sorting experiments. For each pair of parameters (either *C_B_* and *N_int_* or *N_ext_* and *N_int_*) the proportion of the cells that changed phenotype was recorded both for the initially “low *P*” and “high *P*” cells. The difference between the two values δ*S* (for difference in switch rate) indicated whether sorted “low *P*” cells switched more or less frequently or at the same rate than sorted “high *P*” cells. δ*S* is represented on Fig. 5 as a function of *C_B_* and *N_int_* or *N_ext_* and *N_int_* at steps 40 and 100 that correspond to the exponential growth phase of the population. The results showed that both parameter pairs defined three qualitatively distinct regimes. As expected based on the bistable nature of the system, the phenotypic change was highly dependent both on the kinetic parameters and on the noise.

**Figure 6:**
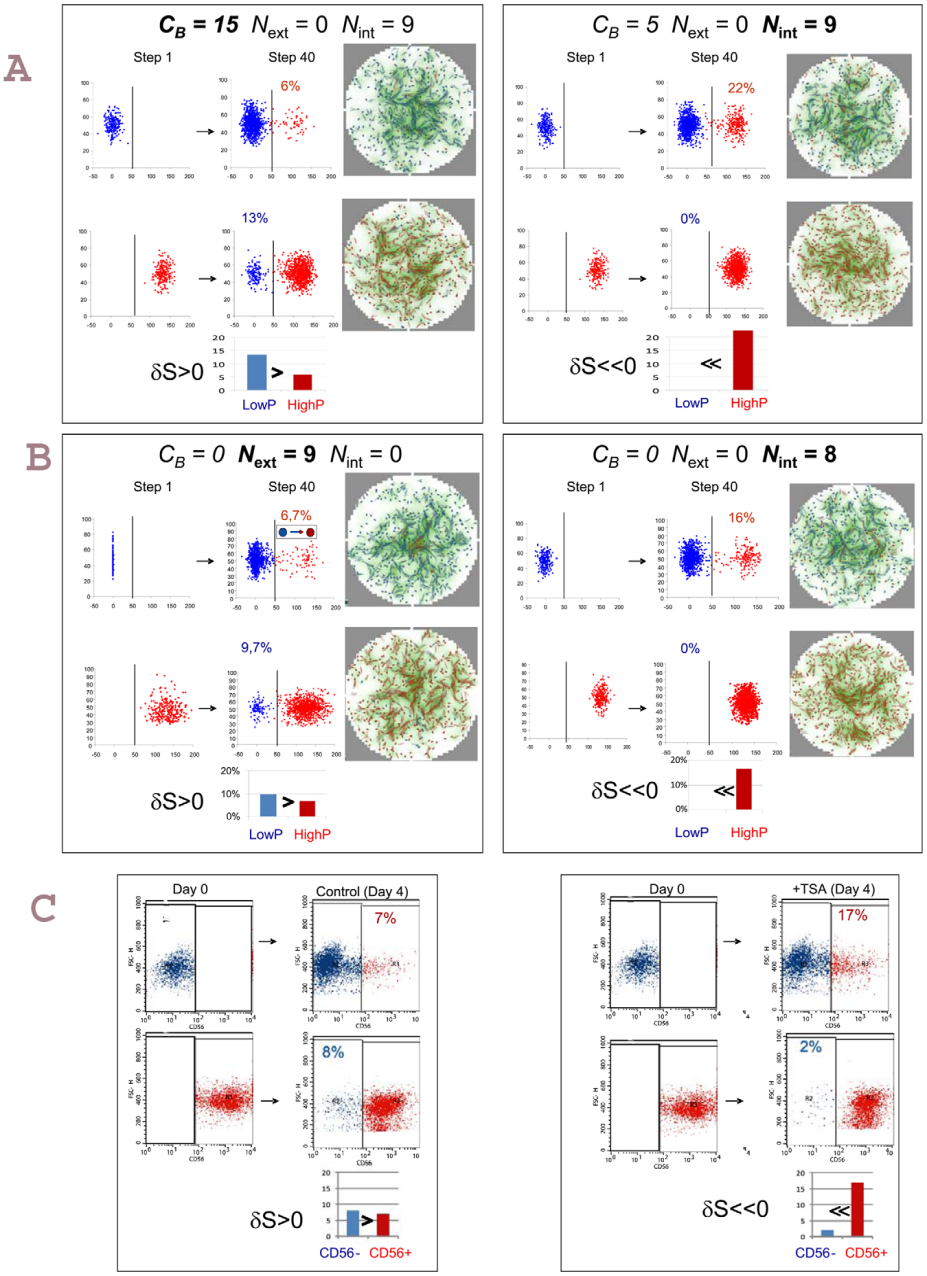
Examples of simulations and effect of TSA. A. Examples taken from the parameter space presented on the Fig. 5. The left panel is an example of strong dependence (*C_B_* = 15) of the kinetic parameter B on local cell density where “low *P*” (blue) cells switch to “high *P*” slower than the opposite: δS>0. The right panel is an example of weak (*C_B_* = 5) dependence of the kinetic parameter B on local cell density where “low *P*” (blue) cells change phenotype easier than the “high *P*” cells: δS <0. The small diagram indicates the fraction of the cells that changed phenotype. Note the density-dependent distribution of the blue and red cells as shown by the virtual “immunochemical” analysis of the cell phenotypes. The graded background is indicative of the local concentration of R decreasing from white to green. B. Examples taken from the parameter space on Fig. 5 with the high context-dependent/low intrinsic (left panel) and high intrinsic (right panel) noise regimes. Note the difference in the conversion rates of the sorted “high *P*” and “low *P*” cells depending on the noise regime as shown by the virtual cytrometrical analysis. The spatial distribution of the “high *P*” (red) and “low *P*” (green) cells is different depending on the noise regime as shown by the virtual immunochemical analysis of the cell phenotypes. C. Trichostatin (TSA) treatment of the living cells reproduces the effect of the high intrinsic noise: the cytometry analysis of sorted and TSA treated CD56− (blue) and CD56+(red) cells compared to non-treated controls shows high conversion rate of the sorted “high *P*”(red) cells compared to “low *P*”(blue) cells (right panel). The control untreated cells show the previously observed conversion rates typical either for strong dependence of parameter B on local cell density or for high context-dependent/low intrinsic noise regime.

When the density dependence of the phenotypic switch was strong (high *C_B_*), the “low *P*” cells converted to “high P” slower than the opposite (Fig. 5). Qualitatively, we observed a similar tendency in living cells. Both the *in vivo* and *in silico* observations are consistent with the fact that the initial cell density at the beginning of the experiment was low; hence, it favoured the “low *P*” state. As a result, “high *P*” cells had the tendency to change their phenotype, whereas “low *P*” cells did not switch. The increasing density during cell proliferation allowed the conversion to the “high *P*” phenotype in regions with high local density (Fig. 5 and Fig. 6A left panel). Therefore, strict density dependence of the bistable phenotype switch is consistent with the observed population dynamics of living cells.

When the local cell density had only weak effect on the *P* production (low *C_B_*) and the intrinsic noise was high, “low*P*” cells converted to “high*P*” phenotype faster than the opposite (Fig. 5 and Fig. 6A right panel). Since the cells tend to a final equilibrium in which the “high *P*” state is more likely than the “low *P*” phenotype and the conversion rate depends only on the noise level. This dynamics may be due to the suppression of the density sensing when the decision to change phenotype is predominantly cell autonomous.

Next we examined the behaviour of the system with constant kinetic parameters (*C_B_*  = 0) and as a function of local cell density- and phenotype-dependent noise (*N_ext_*) and of intrinsic noise (*N_int_*). As expected for a bistable system, when the two noise terms were low no conversion occurred and δ*S* was close to zero. When the context dependent noise was high but the intrinsic noise was low the “high*P*”cells converted to “low *P*” easier than the opposite. This is illustrated on the Fig. 5 and Fig. 6B (left panel).This regime reproduced qualitatively the observations made on living cell cultures. As expected, “low *P*” cells were preferentially located in low-density regions and “high *P*” cells in high-density regions (Fig. 5 and Fig. 6B left panel). The observed asymmetry of the transformation rates was solely due to the double- (density- and phenotype-) dependence of the noise level because the specific regulation of the phenotype determination was identical in all cells and independent of the local cell density. When the intrinsic noise *N_int_* was high and dominated over the effect of the *N_ext_* we observed a dynamic regime with the “high*P*”cells converting to “low *P*” slower than the opposite (Fig. 5 and Fig. 6B right panel). Qualitatively, this behaviour is the opposite of that observed in normal living cell cultures and it was similar to that observed under the conditions when the local density weakly influenced the kinetic parameters of the phenotypic transition (low *C_B_*) with constant intrinsic noise (see above). In addition, this dynamics is reminiscent of a system where the rapid switch to one state and slow relaxation to the initial state was triggered by noise excitation without density sensing described by Kalmar et al. [3].

Based on the simulations it is likely that the apparently paradoxical slow transformation of sorted “low *P*” compared to sorted “high *P*” cells is a logical consequence of the local density-sensing capacity and the fact that the experiment always starts with low initial cell density. The sensing of the local density may occur either by a specific mechanism that targets the activity of myogenic genes or by simply modulating the gene expression noise in a density- and phenotype-dependent way. Since our model provides only qualitative predictions, we cannot directly differentiate the two possibilities.

The simulations suggest that if the capacity of the cells to sense local density is reduced the phenotypic transition of the “low *P*” cells into “high *P*” will be faster than the opposite. This may happen either by lowering the dependence of the transition kinetics (low *C_B_*) or by overruling the context-dependent noise by the high intrinsic noise. To test this prediction, one has either to increase specifically the capacity of the cells to sense local density or increase the intrinsic noise in the cells. It has been reported earlier that Trichostatin A (TSA), a histone deacetylase inhibitor accelerated the differentiation of mouse myoblasts in culture [16]. In order to investigate the changes in δ*S* the CD56+ and CD56− cells were sorted by cell sorter and cultured in the presence of TSA. As shown on the Fig. 6C, as compared to the untreated control, the treatment increased the rate by which CD56+ cells appeared in the culture of initially CD56− cells and substantially slowed down the opposite process. The exact mechanism of action of TSA treatment on the myoblasts is unknown. Nevertheless, it is likely that increasing the level genome-wide of histone acetylation through inhibition of histone deacetylases inducing non-specific chromatin opening would increase gene expression noise due to the random activation of previously repressed genes that would overweight context-dependent noise. Although a specific effect on genes regulating myogenesis cannot be excluded, we can tentatively conclude that a context-dependent noise generating mechanism contributes substantially to the density sensing.

Another conclusion of the simulations is that contrary to our expectations, cell alignment had no effect on the process of phenotypic switch (see Supporting Document S1 and Fig. S3). In fact, suppressing the capacity of the cells to restrict the axis of their migration in dense regions did not modify substantially the dynamics of the phenotypic transition. This aspect will not be discussed further.

## Discussion

Cell differentiation is usually considered a unidirectional process starting with tissue stem cells giving rise to proliferating progenitors that terminally differentiate after several rounds of cell divisions. This view has recently been challenged by observations demonstrating that the heterogeneity observed in pluripotent cells is dynamic and relies on the permanent fluctuation of the cells between different phenotypic states [1], [2], [3], [4]. In all these cases, the cell population represented a dynamic distribution of related states fluctuating between each other. In hematopoietic stem cells, all cells expressed the Sca1 surface marker at varying levels and the sorted low- and high-expressing cells reconstituted a population with the initial distribution slowly [2]. It was proposed that the phenotypic heterogeneity of gene expression level is not due to independent noise in the expression of individual genes, but reflects metastable states of a slowly fluctuating transcriptome that is distinct in individual cells. A model of a noise-driven bi- or multistable system in which different phenotypes correspond to a metastable state of the underlying transcriptional network can account for this type of dynamics. Murine ES cells display a different heterogeneity [3]. These cells are characterized by fluctuations between two clearly different phenotypic states of ES cells; one is stable (“high Nanog”; HN) and the other is unstable (“low Nanog”; LN). The transition between the HN to LN phenotype is stochastic and rare, whereas those from LN to HN are frequent. The observations are consistent with a model with excitable dynamics where the first change is rapid and noise-triggered followed by slow relaxation to the initial state [3].

In our muscle-derived cells, the phenotypic transition appears to differ from the above-described mechanisms but displays some features of both. First, the changes are slow in both directions. Second, the two subpopulations are clearly distinct. In addition, the process is further complicated by the fact that both cell types form wave-like spatial patterns that can potentially modify the local cell density and interfere with the process of phenotypic switch. The observations reported here led us to the conclusion that the dynamics of myoblasts can better be described as a bistable system with the CD56+ and CD56− phenotypes representing the two stable states. Bistability has been observed in cell fate decision and differentiation in various cases [17], [18], [19].

The observations of dynamic phenotypic fluctuations in ES cells led to the proposition that such heterogeneity is a distinguishing feature of the pluripotent state [20], because the capacity to generate heterogeneity is in fact synonymous with the capacity to generate various cell types [21]. The observations reported here suggest that fluctuation between different states may characterise non-terminally differentiated cell types also. We show that every human myoblast can generate at least two phenotypically different, but interconvertible, cell types characterized here by the expression level of the CD56 protein. We show that the fluctuations between the two phenotypic states follow bistable kinetics with slow transition. The proportion of the CD56+/CD56− phenotypes in the population of cells cultured under constant conditions remains approximately stable suggesting that the population is close to equilibrium. We observe that the CD56+ cells have the tendency to be localized in the highly dense regions of the population leading to a partial spatial compartmentalization of the two cell types. Computer simulations were able to reproduce similar spatial compartmentalization only when the cells were able to sense their microenvironment.

The myoblasts form wave-like spatial patterns during population growth. The capacity to form such patterns is a common feature of both CD56+ and CD56− cells. The computer simulations show that these patterns may emerge by the collective behaviour of the cells. The simulations also suggest that the spatial patterns do not contribute substantially to the non-random distribution of the phenotypic forms.

Previous theoretical models suggested that individual cells may gain and loose certain properties depending on whether they localize inside or outside a specific environment [22]. In these models the specific environment existed before the cell's fate decision. Therefore, they cannot explain how the cells are able to reproducibly generate phenotypic heterogeneity even in a homogenous environment. We have proposed previously that cell fate decisions may be made concomitantly with and in tight interaction with the emerging micro-environment [23]. The cell itself constantly contributes to the change of its own environment by secreting and consuming various substances and/or by physically interacting with the neighboring cells. The consequence of these processes is that the phenotypic state of the cells is no longer adapted to the microenvironment they contributed to create. This inadequacy induces a stress response, increases cell-intrinsic fluctuations and encourages the cell to explore alternative possible phenotypic states until equilibrium is restored. Our previous findings on the uneven spatial distribution of stem-like cells in mouse myoblast cultures suggested that adaptation to the local microenvironment may constitute the first step in the emergence of a new cellular phenotype [1]. More recently, Snijder et al. has extended our initial observations by revealing correlations with specific cellular states that are defined by the population context [5]. The authors demonstrated that virus infection, endocytosis and membrane lipid composition are determined by the cellular microenvironment, mainly by local cell density. The observations reported here go beyond the demonstration of the correlation between the “ecological context” and phenotype and suggest simple principles that can reconcile widespread stochastic fluctuations of gene expression on one hand and an ordered sequence of events resulting in stable cellular states with defined spatial distribution.

Stable phenotypic states are frequently represented as “high dimensional attractors” of the transcriptome in the “potential energy landscape” or in the “noise landscape” [9], [24]. In this contemporary reformulation of the “epigenetic landscape” metaphor proposed by Waddington the landscape of high dimensional attractor states inferred from the gene regulatory network architecture is necessarily intrinsic to the cell. The transition between two states is triggered by the noise of the transcriptional regulatory network [24]. Recent observations on adaptive attractor selection in bacteria provided direct experimental support to this hypothesis [25]. Our work extends this view by suggesting that the “epigenetic landscape” is not intrinsic to the cell and it is not stable in time but dynamically changing. Its exact shape is determined by all participant cells through the interplay between the fluctuating intrinsic state of individual cells and the interactions between the neighbouring cells that form the microenvironment. This interpretation is similar to conceptual models that tend to abandon the classical assumption of a strict hierarchy during differentiation and understand cell differentiation as a dynamic process of “isologous diversification” or autostabilization of stochastic processes [26], [27], [28], [29].

In vitro cell cultures like those studied here are frequently used to investigate features of *in vivo* tissues. Although our experimental system does not reproduce with precision the organization of the muscle tissue *in vivo*, the observations reported here also offers some clues for interpreting some observations made *in vivo*. When satellite cells are activated by muscle damage, they undergo rapid cell divisions before differentiating to form myofibers. However, a fraction of the cells returns to the quiescent cell pool. The choice between the two fates is reminiscent of the *in vitro* situation. A recent study has shown that both autocrine and paracrine feedback mechanisms act both *in vivo* and *in vitro* to bring about this dichotomous fate decision [30]. It is possible that the dynamics of this process in the complex *in vivo* situation also follows a noise driven bistable logic identified in our *in vitro* cellular system.

## Materials and Methods

### Human primary skeletal muscle cell culture

We used previously described primary muscle cells [11]. Cells were cultured in growth medium composed of one volume of medium 199 (Gibco) and one volume of DMEM 4,5 g:l glucose (Gibco) supplemented with 20% fetal calf serum (Gibco) at 37°C in a humid atmosphere containing 5%CO_2_. For all experiments, the myoblasts used were between 25-35 population doublings.

### FACS analysis

For flow cytometric analysis, after 4 or 7 days in growth medium, 2×10^5^ cells were detached from the surface of the culture dish using TrypLE select (Gibco), resuspended in PBS containing phycoerythrin-conjugated anti-CD56 (Milteny, clone AF12-7H3) antibody diluted 1 to 15 for 10 min at 4°C. Cells were washed and analysed using FACSCalibur (BDbiosciences). Phycoerythrin was detected off the 488 nm line using logarithmic amplification. Each acquisition file included at least 5000–10000 events. Sample incubated with irrelevant isotype-matched (Milteny, clone IS5-21F5) served as control for background fluorescence. A forward scatter (FSC) threshold was set to eliminate debris from list mode data and for each sample.

### Isolation of CD56+ and CD56- cells

Cell-sorting was performed using Beckman Coulter MoFlo. Cells (5×10^6^–1×10^7^cells) were detached and incubated with PE-CD56 (see FACS analysis). PE was detected off the 488 nm line using logarithmic amplification. Sample incubated with irrelevant isotype-matched antibody (Milteny, clone IS5-21F5) served as control for background fluorescence. After determination of the purity of the two types of populations (only at least 98% pure CD56+ or CD56- fractions were used), cells were plated into 6-well plates (2000–3000 cells/cm^2^) or into 96-well plates in the presence of proliferation medium (500–3000 cells/cm^2^ or one cell by well). Cell cloning was performed after similar labelling procedure. The individual cells were plated into 96-well plates in proliferation medium.

In the TSA experiment CD56+ and CD56− cells were plated at a density of 2.8×10^4^ cells per well into 6-well plates. Trichostatin A (Sigma) added to the growth culture medium at 70 nM for 4 days.

### Immunostaining, microscopy and image processing

Cells were washed with phosphate-buffered saline (PBS) (Gibco) and fixed with 4% paraformaldehyde for ten minutes, rinsed two times with PBS, blocked with 2% goat serum and incubated for one hour with PE-conjugated antibody anti-CD56 (Miltenyi; Ref: 130-090-755; dilution 1/15). Nuclei of cells were counstained by DAPI (Invitrogen). For labeling the cell membrane, we used CellTrace^TM^ CFSE Cell proliferation kit (Molecular probes). Fluorescent images were acquired using the acquisition software Cartograph (Microvision, Courcouronne, France) controlling an inverted IRDM microscope (Leica) mounted with a motorized stage and a MicroPublisher 3.3 camera (Qimaging). The total surface of each well was scanned in both fluorescence (Blue and Red) using a 10× objective (192 images: 12×16 rectangle). Mosaics of images were exported in tif format and treated with imageJ (http://rsb.info.nih.gov/ij/). The analysis was based on the same philosophy as previously for the study of the stem cell-like side population (SP) cells in the mouse myogenic cell line [31]. Cells were segmented using a threshold for the blue channel (Dapi) and by collecting intensity information from the Red channel (CD56). Coordinates for each cell with the CD56 intensity signal was exported in text for the spatial analysis. The R program was used for the statistical spatial analysis using homemade scripts as well as the open source SpatStat Package [32] used for calculating the nearest neighbors distances.

### Superoxide detection in cultured myoblasts

Generation of superoxide anions by myoblasts in culture was assessed using DHE (Dihydroethidium, Invitrogen, Cergy-Pontoise, France). DHE is predominantly oxidized by short-lived superoxide anions to ethidium, which intercalates within the cell's DNA and generates a nuclear fluorescence in cells. The myoblasts were grown on glass cover slips (VWR, Fontenay-sous-Bois, France). DHE (10 µM final concentration) was added to the cell culture medium, and cultures were incubated for 30 min at 37°C. The medium was then removed and cultures were rinsed once with PBS. Cells on cover slips were fixed with paraformaldehyde (4%, pH 7.5) for 10 min at room temperature. They were then rinsed once in PBS, stained with the nuclear fluorescent dye TO-PRO-3 (Invitrogen, 1∶1000 diluted with PBS) for 5 min and washed with PBS. Glass cover slips were mounted on slides with Fluoromount (Clinisciences, Montrouge, France). Images were captured using a Leica DMRE confocal microscope with laser excitation at 514 nm and emission measurements using a 580/620 nm band pass filter.

### Description of the model

The computer simulation was performed using the Netlogo language, specifically designed to make simple agent-based models (http://ccl.northwestern.edu/netlogo/). The code is available on request (stockho@genethon.fr or paldi@genethon.fr).

The basic assumptions of the standard model were as follows: the cells were able to divide, migrate and die. The cell cycle length and migration velocity were carefully calibrated of the basis of the time-lapse records.

#### Bistable switch model of phenotypic transition

In the model, each cell is an autonomous agent. We hypothesized that the phenotype is contingent on the accumulation of a substance *P* which can represent one or several proteins in the cell. This approach made possible the direct comparison of the simulated cellular phenotypes described by *P* in the model and the level of CD56 protein that describes the phenotype of the living cells. The actual level of *P* is calculated at each step of the simulation as a function of the production and degradation rates. It is described by the equation:
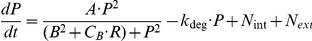



The first term of the equation describes the production of phenotype “*P”* by the cell. It is assumed to be non-linear and modelled using the Hill function with a Hill parameter n = 2. The second term describes the degradation of “*P”*, presumed to be linear with a rate constant *k_deg_*. The non-linear production and linear degradation together ensure a dynamic behaviour with two stable states. Phenotypic stability is achieved when the production and degradation rates are equal and the amount of *P* in the cell remains stable. There are three such states; one with high and the second with low *P* level. These two states constitute attractor states. The third equilibrium is unstable.

The parameter *A* determines the maximal rate of *P* production. The form of the sigmoid curve, that is how rapidly the maximal rate is reached as a function of *P*, is determined by the parameter *B*. In other terms, the proportion of “low *P*” and “high *P*” cells in the population at equilibrium depends on *B* providing the degradation rate remains unchanged. The dependence of *B* on the local cell density is achieved using the term *C_B_R*. *C_B_* is a parameter that determines how strong is this dependence and the variable *R* is an indicator of local cell density. The actual value of *R* is calculated at each step for each region of the virtual Petri dish (called “patch” in Netlogo) as the equilibrium between the quantity that is consumed by the cells and the constant diffusion of the substance from the surrounding culture medium. The initial concentration of *R* is 100 everywhere in the culture, but it gradually decreases in the regions of high cell density if the diffusion cannot compensate the utilization. Therefore, *C_B_R* is high (if *C_B_*>0) in regions of low cell density and favours “low*P*” phenotype by increasing the denominator of the Hill-function and decreases with high cell density making the “high*P*” phenotype more likely (Fig. 4C). In other terms, the actual local cell density modifies the kinetics of phenotypic transition of the cell at each step.

However, the transition between the two states is impossible without stochastic fluctuations of the production and degradation of *P*. We introduced two noise terms in the equation: *N_int_* describes the cell-intrinsic noise that is calculated at each step as a normally distributed random variable with average µ*_Nint_* = 0 and fixed standard deviation σ =  *N_int_*.

The last term *N_ext_* describes the noise generated by the microenvironment of the cell. *N_ext_* is also a normally distributed random variable with average µ*_Next_*  = 0. It is calculated for each cell at every simulation step. Hence, the actual value of the extrinsic noise in a cell fluctuates around 0 and can increase or decrease the level of *P* in the cell. We assumed that the noise amplitude increases with the local cell density, but decreases as a function of *P* level in the cell which is described by a function *N_ext_* derived from a hyperbolic paraboloïd (f(x,y) = xy):
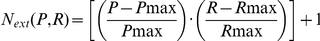



This function determines a surface depicted on the Fig. S2. As a result, in “low *P*” cells the amplitude of the noise is maximal if the local cell density is high, while in “highP” cells the noise is the highest at low cell density. The noise is minimal in “low *P*” cells at low density and “high *P*” cells at high density. In other terms, the extrinsic noise depends on the match between the internal state of the cell and its local environment (Fig. S1).

### Statistical analysis

For all statistical calculations we used the R package [32] version 2.8.0. (http://journal.r-project.org/).

## Supporting Information

Document S1 Supporting Text describing: 1. Simulations showing no impact of cell aligment on the phenotypic switch in the model 2. Methylation analysis 3. Supplementary Reference 4. Supplementary Methods for i. Analysis of cell migration using time-lapse videomicroscopy ii. Analysis of cell proliferation and iii. DNA extraction and DNA methylation analysis of the CD56 gene.
(0.04 MB DOC)

Figure S1 Quantification of the migration and proliferation properties of the human cells for computer simulation. (A) Time lapse experiments for the determination of cell migration characteristics. A low cell-density culture with the cell velocity vectors of a selection of moving cells are shown on the left panel. The same culture 48 hours later with the velocity vectors of the moving cells is shown on the right panel. Note the tendency of the velocity vectors to become parallel in the high cell density regions. (B) Example of cell trajectories in a low-density culture as detected by time-lapse video microscopy and cell tracking (on the left panel). Right panel: a histogram showing the exponential distribution of the cumulative velocity magnitudes on the basis of 16000 individual velocity values. (C) Measure of proliferation rate of the cells using PKH26. The parental population is shown on the left panel. The distribution of the fluorescence after three days of culture and the deconvolution analysis (right panel) indicate that the average cell cycle length is between 20 to 24 hours. This value was also confirmed by the tracking of individual cells on time-lapse records.
(1.87 MB TIF)

Figure S2 Representation of the function describing the context-dependent noise Next as a function of the local density and the phenotype defined by the intracellular level of P. The function follows the general form of a hyperbolic paraboloid, f(x,y) = xy.
(0.28 MB TIF)

Figure S3 Analysis of the parameter space in the model with random cell migration and without cell alignment. Note that at steps 40 and 100 the behaviour of the system in the parameter space defined by the two noise terms is identical to that seen in the model with cell alignment (Fig. 5).
(1.86 MB TIF)
