# Supplementary material for: Bistable Cell Fate Specification as a Result of Stochastic Fluctuations and Collective Spatial Cell Behaviour
Source: PLoS One. 2010 Dec 28;5(12):e14441. doi: 10.1371/journal.pone.0014441 (PMC3010982; doi:10.1371/journal.pone.0014441)
Supplement: Document S1 — Supporting Text describing: 1. Simulations showing no impact of cell aligment on the phenotypic switch in the model 2. Methylation analysis 3. Supplementary Reference 4. Supplementary Methods for i. Analysis of cell migration using time-lapse videomicroscopy ii. Analysis of cell proliferation and iii. DNA extraction and DNA methylation analysis of the CD56 gene. (0.04 MB DOC) [file pone.0014441.s001.doc]

**Supporting Document S1**

**No impact of cell alignment on the phenotypic switch in the model.**

We simulated the effect of spatial pattern formation on the distribution and transformation kinetics of the cells. The effect of cell alignments was estimated by comparing the results obtained in simulations in the context-dependent model to those obtained with a control model in which the capacity of the cells to align and form spatial patterns was suppressed and replaced by simple random migration while the features of the density dependent phenotypic transition were conserved. The supplementary Fig.S2 shows the part of the parameter space defined by the two noise terms. The results were very similar to those obtained in the model with cell alignment. Three regimes were observed. As expected, “high *P*” expressing cells are preferentially located in highly dense regions and “low *P*” cells in low-density regions in both models. Therefore, the impact of cell alignment on the phenotypic conversion is not critical. A slightly stronger correlation between the cell density and the phenotype was observed in the absence of wave-like pattern formation suggesting that rapidly emerging cell alignments may act as a stabilizing force by reducing the frequency by which the cells change phenotype and by reducing the rate at which the system moves to equilibrium. This point was not further investigated. Similar results were obtained when the part of the parameter space defined by *C_B_* and *N_int_* was investigated (not shown).

**Methylation analysis**

In order to exclude the possibility that action of TSA is simply limited to the activation of the CD56 gene by changing its epigenetic state we analyzed the mCpG methylation of the gene’s regulatory sequences using the bisulfite sequencing method. The analysis revealed that the sequence is demethylated both in CD56- and CD56+ cells. This indirectly suggests that the increase of CD56 expression is not a simple result of a specific epigenetic derepression of transcription of this gene.

**Supplementary references**

Shen H, N. A., Sugimoto J, Sakumoto N, Oda T, Jinno Y, Okazaki Y. (2006). Tissue specificity of methylation and expression of human genes coding for neuropeptides and their receptors, and of a human endogenous retrovirus K family. Journal of Human Geneti*cs* 51, 440-450.

**Supplementary Methods**

**Analysis of cell migration using time-lapse videomicroscopy.**

Records of cell migration were done using a Zeiss Axiovert 100M confocal microscope. The cells were maintained under controlled CO2 atmosphere and temperature. Bright field images were recorded every 10 minutes up to 96 hours. Image acquisition was done at high resolution (1024*6*1024 pixels) with aid of Zeiss LSM 510 software for PC. Migration velocities were calculated using a ‘‘Manual Tracking’’ plug-in (Fabrice Cordelie`res, Institut Curie, Orsay, France) of ImageJ software (<http://rsb.info.nih.gov/ij/>). The data were exported and analysed in Excel.

**Analysis of cell proliferation.**

Cell cycle length and variation was determined by cytometry after cell membrane labelling with PKH26 (Sigma Aldrich). The cells were labelled by the dye according to the manufacturer’s instructions and cultured for several days. The distribution of the fluorescence was determined by cytometry. The data were analysed by the ModFit software.

**DNA extraction and DNA methylation analysis of the CD56 gene.**

Sorted CD56 positive and negative cells were used. DNA was extracted using QIAGEN Genomic tip (QIAGEN, Hilden, Germany) according the manufacturer’s protocol.

Methylation status of the CD56 gene was investigated using sodium bisulfite sequencing. Sodium bisulfite treatment of genomic DNA and following DNA purification were carried out using EpiTect (QIAGEN) according the manufacturer’s protocol. The DNA fragments covering the promoter region (Region A) and intragenic region (Region B) of CD56 gene were amplified by (semi-) nested PCR using the listed sets of primers. The primer sequences of Region A were obtained from previous paper (Shen H, 2006), and the primers of Region B were newly designed. The PCR reaction products were cloned into pGEM-Teasy vector (Promega, Medison, WI), and clones randomly picked from each of two independent PCRs were sequenced to determine the presence of methylated cytosines. Primers used: Region A First forward: GGAAGGTTGGGTAGTAGGAG; Reverse: CCTAAAAACAAACAATTACCAAAC; Region A Second forward: GAAATTTTAGTTTTTTTAGGGAG; Reverse: CCCCAAACAACAATAAAAAAAAAC; Region B First forward: TGGAAGAATTAGGAAAGAGTTGA; Reverse: CCCCAAACAACAATAAAAAAAAAC; Region B Second forward: TGGAAGAATTAGGAAAGAGTTGA; Reverse: TGGAAGAATTAGGAAAGAGTTGA.
