# Supplementary figures and images for: Bistable Cell Fate Specification as a Result of Stochastic Fluctuations and Collective Spatial Cell Behaviour
Source: PLoS One. 2010 Dec 28;5(12):e14441. doi: 10.1371/journal.pone.0014441 (PMC3010982; doi:10.1371/journal.pone.0014441)

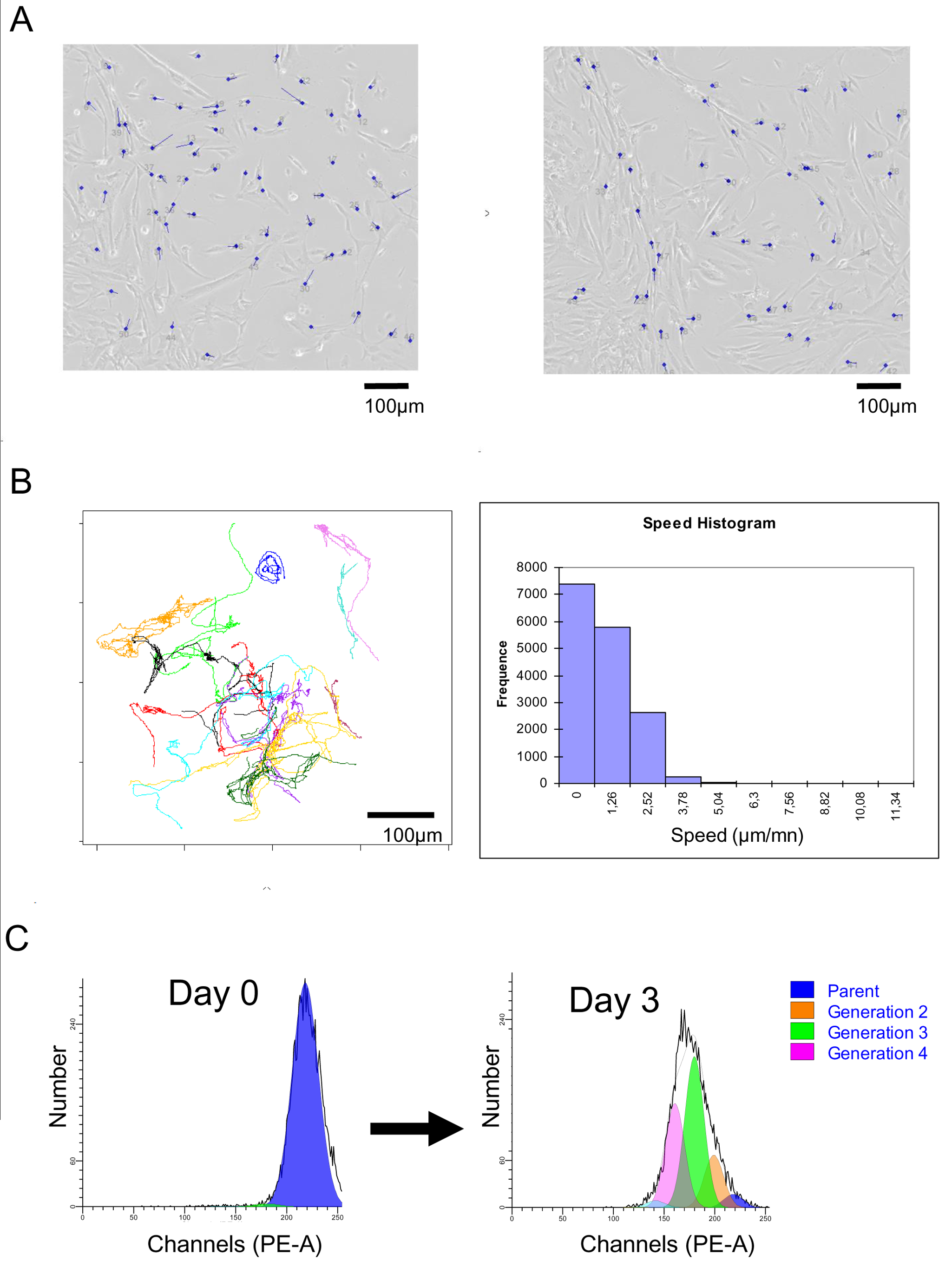

Supplement: Figure S1 — Quantification of the migration and proliferation properties of the human cells for computer simulation. (A) Time lapse experiments for the determination of cell migration characteristics. A low cell-density culture with the cell velocity vectors of a selection of moving cells are shown on the left panel. The same culture 48 hours later with the velocity vectors of the moving cells is shown on the right panel. Note the tendency of the velocity vectors to become parallel in the high cell density regions. (B) Example of cell trajectories in a low-density culture as detected by time-lapse video microscopy and cell tracking (on the left panel). Right panel: a histogram showing the exponential distribution of the cumulative velocity magnitudes on the basis of 16000 individual velocity values. (C) Measure of proliferation rate of the cells using PKH26. The parental population is shown on the left panel. The distribution of the fluorescence after three days of culture and the deconvolution analysis (right panel) indicate that the average cell cycle length is between 20 to 24 hours. This value was also confirmed by the tracking of individual cells on time-lapse records. (1.87 MB TIF) [file pone.0014441.s002.tif]

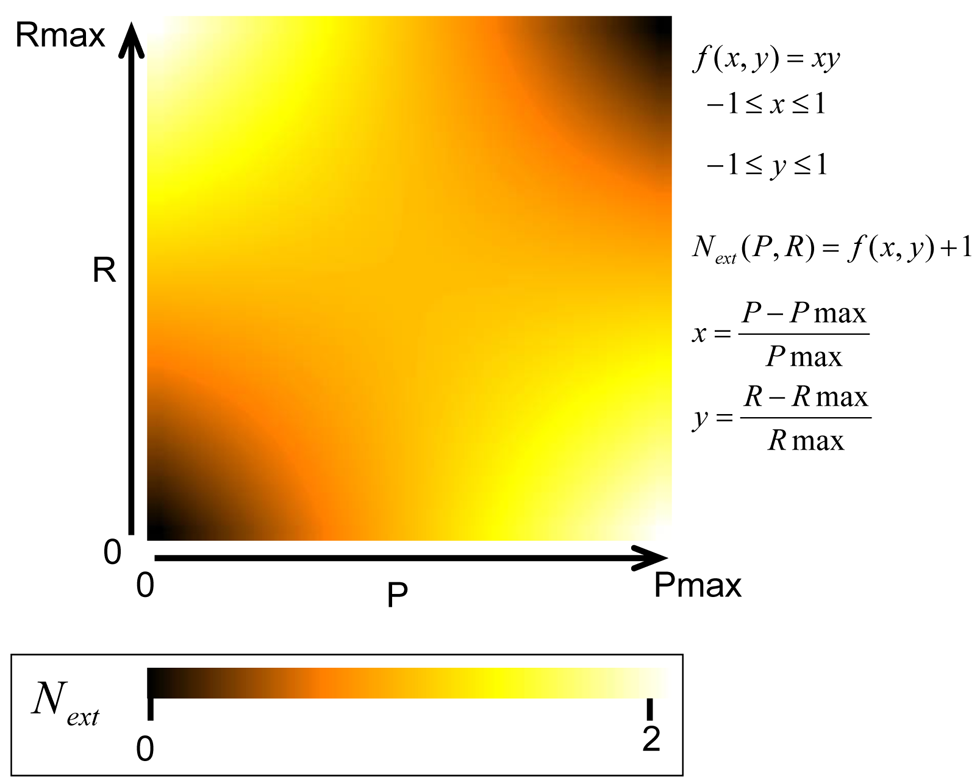

Supplement: Figure S2 — Representation of the function describing the context-dependent noise Next as a function of the local density and the phenotype defined by the intracellular level of P. The function follows the general form of a hyperbolic paraboloid, f(x,y) = xy. (0.28 MB TIF) [file pone.0014441.s003.tif]

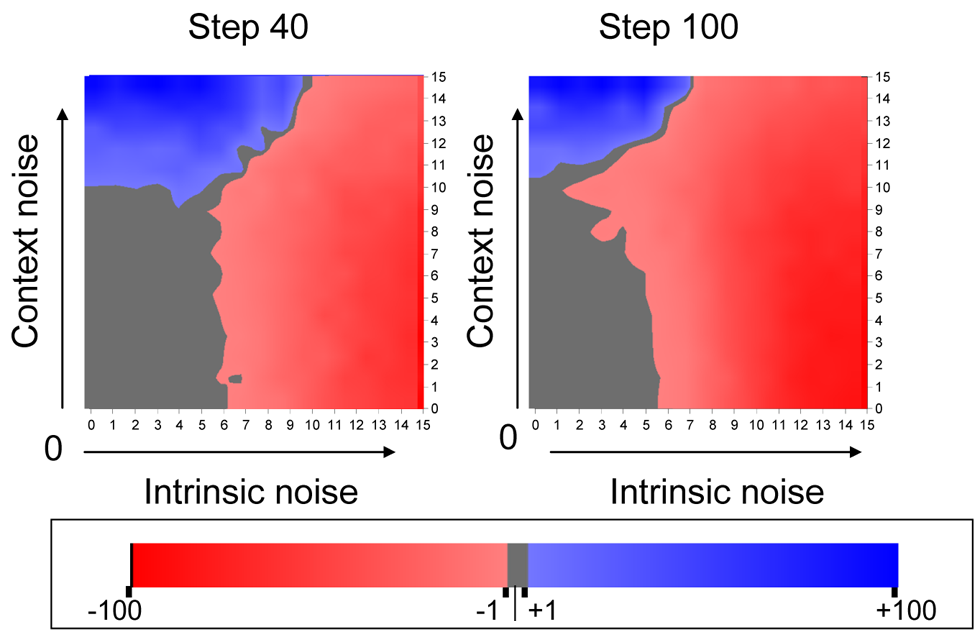

Supplement: Figure S3 — Analysis of the parameter space in the model with random cell migration and without cell alignment. Note that at steps 40 and 100 the behaviour of the system in the parameter space defined by the two noise terms is identical to that seen in the model with cell alignment (Fig. 5). (1.86 MB TIF) [file pone.0014441.s004.tif]
